# Model-based aversive learning in humans is supported by preferential task state reactivation

**DOI:** 10.1126/sciadv.abf9616

**Published:** 2021-07-28

**Authors:** Toby Wise, Yunzhe Liu, Fatima Chowdhury, Raymond J. Dolan

**Affiliations:** 1 Max Planck UCL Centre for Computational Psychiatry and Ageing Research, University College London, London, UK.; 2 Wellcome Centre for Human Neuroimaging, University College London, London, UK.; 3 Division of the Humanities and Social Sciences, California Institute of Technology, Pasadena, CA, USA.; 4 State Key Laboratory of Cognitive Neuroscience and Learning, IDG/McGovern Institute for Brain Research, Beijing Normal University, Beijing, China.; 5 Chinese Institute for Brain Research, Beijing, China.; 6 Queen Square MS Centre, Department of Neuroinflammation, UCL Queen Square Institute of Neurology, London, UK.

## Abstract

Harm avoidance is critical for survival, yet little is known regarding the neural mechanisms supporting avoidance in the absence of trial-and-error experience. Flexible avoidance may be supported by a mental model (i.e., model-based), a process for which neural reactivation and sequential replay have emerged as candidate mechanisms. During an aversive learning task, combined with magnetoencephalography, we show prospective and retrospective reactivation during planning and learning, respectively, coupled to evidence for sequential replay. Specifically, when individuals plan in an aversive context, we find preferential reactivation of subsequently chosen goal states. Stronger reactivation is associated with greater hippocampal theta power. At outcome receipt, unchosen goal states are reactivated regardless of outcome valence. Replay of paths leading to goal states was modulated by outcome valence, with aversive outcomes associated with stronger reverse replay than safe outcomes. Our findings are suggestive of avoidance involving simulation of unexperienced states through hippocampally mediated reactivation and replay.

## Teaser

The human brain simulates the outcomes of actions to enable avoidance of danger.

## INTRODUCTION

Simulation of future states has gained increasing attention as a mechanism supporting decision-making, particularly in the absence of direct experience (*1*). This is thought to be supported by neural replay, evident in observations that hippocampal place cells and adjacent place fields reactivate in a forward or reverse sequence, reflecting future or past trajectories (*2*, *3*). Notably, paths leading to aversive outcomes are reported to show preferential replay during avoidance behavior (*4*), implicating a simulation of these paths as a neural signature of harm avoidance. Intriguingly, one recent proposal suggests that the brain’s ability to simulate both experienced and hypothetical outcomes might provide for a mechanistic understanding of phenomena such as worry and rumination (*5*–*7*).

In humans, simulation of individual rewarded states has been found to support planning and inference (*8*–*11*) as well as updating of reward values (*12*). We refer to this simulation process as “reactivation,” as it entails reinstating an aspect of a previously encountered state’s neural representation (for example, perceptual qualities associated with the stimulus). This reactivation can reflect simulation of states that may be distant from an individual’s current location. This process allows for a simulation of extended sequences of actions that extend beyond the current state, potentially supporting prospective decision-making (*8*). Recent human neuroimaging findings show that, across a variety of tasks, reactivation of singular events can occur in sequence in a manner reminiscent of rodent hippocampal replay (*13*–*18*). Collectively, these studies suggest that state reactivation is a motif of human model-based learning and decision-making. While a recent study demonstrated reactivation of potential outcomes during avoidance (*19*), it is unclear as to what extent this is related to model-based avoidance, which is critical for successful avoidance of danger and, arguably, has a strong relevance to common symptoms of mood disorders (*5*, *20*–*22*).

Here, our focus is on the contribution of reactivation and replay (i.e., sequential reactivation) to human avoidance behavior. We used magnetoencephalography (MEG) to index states while participants engaged in an aversive learning task that promotes avoidance decisions, based on inference about learned aversive state values. Specifically, we ask whether reactivation and replay of task states underpin model-based avoidance and aversive value updating. We use the term “model-based” to refer to decisions where option values are determined by exploiting an internal model of the task, rather than using stimulus-outcome associations learned through experience. Our results indicate that replay supports model-based aversive learning through simulation of states that have not been the object of direct experience. A simulation of aversive states offers the intriguing possibility that this type of process might be relevant for understanding a range of phenomena in psychopathology, including ruminative negative thought patterns characteristic of mood disorders (*7*).

## RESULTS

### Participants adaptively use model-based control to facilitate avoidance

**Fig. 1:**
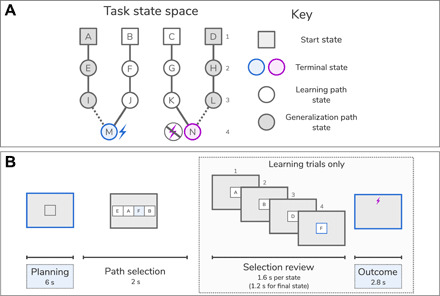
Task design. (**A**) Illustration of task states and transitions. Participants navigated a map comprising 14 states (labeled A to N), each represented by a unique visual image. On learning trials, participants chose between two learning paths (B > F > J > M, C > G > K > N), which, at the terminal state, led to a shock or safe outcome according to a drifting shock probability determined by a random walk. On generalization trials (28%), participants chose between the two generalization paths (A > E > I > M and D > H > L > N). For choices on these paths, the associated outcomes were not shown to participants to obviate learning. (**B**) Trial procedure. Each trial began with a 6-s “planning” phase when participants viewed a colored square representing a trial type (one color indicating learning trials and another indicating generalization trials). The subjects were instructed to think about the sequence of states that they wished to select. The participants then selected a sequence of states that took them from a starting state to one of the final states (the “state selection” phase). The participants were presented with a state from an array of four presented images that included valid state(s) (i.e., states to which participants could validly transition from the current selected state) as well as randomly selected invalid states. On learning trials, after selecting a path, the entire sequence trajectory was shown sequentially (the “selection review”). At the final state, the participants saw either a shock icon (indicating an upcoming shock) or a crossed shock icon (indicating safety). Outcomes (both shock and safety) were accumulated, and three were randomly administered at the end of each block of 20 trials. On generalization trials, the trial ended after state selection without playback of the path, with the participants told that the hidden outcomes would accumulate and be administered upon completion of the entire task, unlike outcomes from the learning trials that were administered at the end of each block.

**Fig. 2:**
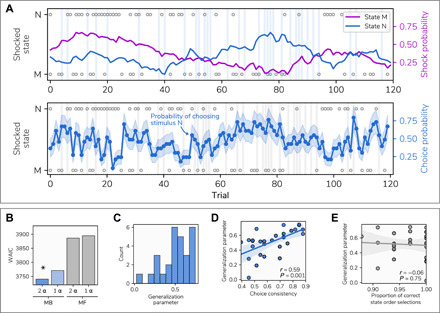
Behavior. (**A**) Trial outcomes and participants’ responses. Top: Purple and blue lines indicate shock probability for the final states of each learning arm; these followed independent (moderately anticorrelated) random walks, such that each state was safest on an approximately equal number of trials. Blue vertical bars represent generalization trials. Bottom: The blue line represents the proportion of participants choosing option N, with shaded area representing the SE. Gray circles indicate which state was shocked on a given trial, with those at the top indicating a shock for state N and the bottom indicating a shock for state M. (**B**) Model comparison (see Materials and Methods) demonstrated superior performance of a model incorporating asymmetric updating from shock and safety outcomes, as well as model-based inference on generalization trials. MF represents model-free control; MB represents model-based inference. α refers to the learning rate parameter, either dependent on outcome valence (2 α) or the same for both shock and no shock (1 α). * indicates the model with the lowest WAIC. (**C**) Estimated generalization parameter values across participants. Values of 0 represent no model-based inference (i.e., choosing randomly on generalization trials), while a value of 1 indicates choices fully consistent with that expected if these were made on the basis of a learned value. (**D**) Correlation between generalization parameter values and choice consistency between adjacent learning and generalization trials, a model-agnostic approximation of a tendency to use model-based inference. The strong relationship indicates that this parameter provides a valid representation of this behavior. (**E**) Nonsignificant correlation between generalization parameter values and the number of errors made on generalization trials across participants (where participants failed to enter a correct sequence of states), showing that low generalization parameter values do not reflect poor knowledge of task structure. If this were the case, then more errors would be associated with less generalization.

Twenty-eight participants (20 female and 8 male) completed an aversive learning task while we acquired simultaneous neural data using MEG. The task space consisted of 14 discrete states, each represented by unique visual images. Participants navigated from start to terminal states (Fig. 1A), where the latter was associated with a drifting probability of an electric shock. Shock probabilities were designed to be moderately, but not perfectly, anticorrelated (*r* = −0.57; Fig. 2A). This ensured that one option was generally preferable, while requiring a representation of those outcome types (shock and safety) for each terminal state. The task space included two arms (referred to as generalization arms) that terminated at the very same state as one of the other two arms (referred to as learning arms).

On most trials, participants had to choose between the two learning arms and observed an outcome screen upon reaching the terminal state. Rather than administering shocks during task performance trials, the outcome types accumulated over the course of each block. Subsequently, at the end of a block, three randomly selected outcomes were administered, a design feature that avoids contamination of the task phase by processing of shock-related signals, or movements in response to the shock. Crucially, on a subset of trials (28% of trials, referred to as generalization trials), the participants chose between two generalization paths. The outcome for a generalization path was never shown, obviating learning through direct experience within these arms. Instead, the next trial started immediately. The subjects received prior instruction that the hidden outcomes from these generalization trials would nevertheless accumulate and be delivered at the end of the task, ensuring that decisions on generalization arms promoted a model-based inference in relation to a hypothetical final state.

To examine the computational processes underlying task avoidance behavior, we fit a reinforcement learning model that updates aversive value on each trial according to an outcome prediction error (*23*). Learning was weighted via separate learning rates for better- and worse-than-expected outcomes, allowing an asymmetry in learning previously observed in these types of aversive learning tasks (*24*, *25*). This provided a better fit to the data, as assessed using the Watanabe-Akaike information criterion (WAIC) (*26*), than a variant model relying on symmetric updating (asymmetric WAIC = 3744.55, symmetric WAIC = 3770.09; Fig. 2B). As in previous work, the participants varied in their tendency to learn from safety versus punishment (*27*), though we found no consistent bias toward learning from danger when comparing learning rates for shock and safety outcomes [*t*(27) = 0.85, *P* = 0.40], as observed in our prior studies (*24*, *25*).

We incorporated into our model an additional mechanism that allowed learned values for the two end states to inform decisions on generalization trials, which rely solely on the exercise of model-based inference (see Materials and Methods). The degree of model-based inference was represented by a free parameter *G*, where a value of 0 entailed choices on generalization trials were random and a value of 1 entailed choice probability was identical to that for learning trials. Model comparison indicated that models including model-based inference outperformed models that chose randomly on generalization trials (WAIC for best-performing model without generalization = 3888.18), consistent with participants deploying a model-based inference (Fig. 2B). A closer examination of the estimated values for *G* revealed substantial variability across participants (Fig. 2C). This parameter was positively correlated with a purely behavioral (model agnostic) index of model-based choice (i.e., the choice consistency between generalization trials and preceding learning trials; *r* = 0.59, *P* = 0.001; Fig. 2F) but not with a measure of task transition knowledge (*r* = −0.06, *P* = 0.75; Fig. 2E), consistent with it reflecting use of a model-based strategy rather than a readout of accuracy in deployment of task knowledge (see Supplementary Results). While our task was not specifically designed to maximize the benefit of using model-based control, we found a moderate, but nonsignificant, negative correlation between *G* values and number of shocks received (*r* = −0.32, *P* = 0.09), providing tentative evidence that greater use of model-based planning facilitated avoidance.

### Model-based planning is associated with replay of task states

**Fig. 3:**
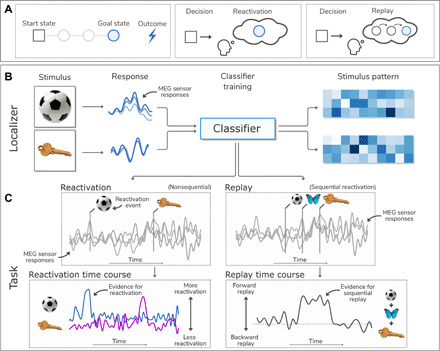
Overview of replay and reactivation analysis approach. (**A**) Illustration of approach, focusing on planning. Participants are asked to navigate from a start state to a goal state of their choice. We assess evidence for reactivation (isolated simulation of individual states) and replay (sequential reactivation of a series of states). (**B**) Cartoon illustration of the classifier training procedure. Stimuli are shown to the participant, producing a stimulus-specific neural response. A classifier is then trained to label each response pattern, as recorded using MEG, with its correct stimulus label by learning the multivariate pattern of response characteristic of each stimulus. (**C**) In the task, this classifier is then asked to ascertain the likelihood of each stimulus being reactivated at each time point in relation to data recorded during outcome and planning phases, a time period when no actual stimuli are shown to the participant. If the classifier indicates that the signal is similar to that associated with a particular training stimulus, then we infer that this stimulus is likely being reactivated. Reactivation can either occur in isolation or in a sequential pattern representing actual stimulus sequences in the task state space, which we term replay. This procedure generates a time course of reactivation likelihood for each stimulus, and the sequenceness analysis uses these time courses to generate a time course of evidence for forward and backward sequenceness.

We first asked whether, at decision time, there was evidence for neural reactivation of task terminal goal states linked to avoidance decisions. We used a temporal generalization approach, as used previously (*10*), to train a classifier to discriminate between the terminal states of either arm (Fig. 3). To ensure that trained classifiers were unbiased by the state’s position or value in the task itself, we used classifiers trained on a pretask functional localizer wherein participants repeatedly viewed images in a random order. These images were first shown before learning any sequential or value relationships for classifier training and only later used in the subsequent main task. Thus, these classifiers captured neural responses unique to images that represented discrete states, allowing us to differentiate between the terminal states of each arm. Critically, we could apply this classifier to task data to ask whether reactivation predicted behavior (i.e., how well the classifiers predicted the chosen path at the outcome phase). Thus, if predictions from the classifier align with an actual decision, then we could infer that the perceptual representation of a selected stimulus was goal relevant at that time.

While the functional localizer included all task stimuli, for the analysis of terminal-state reactivation, we trained a binary classifier on these terminal states alone. Here, we iteratively trained the classifier at 10-ms (one sample) intervals up to 800 ms after stimulus onset. Subsequently, we applied the classifier across task periods of interest at 10-ms intervals, producing a two-dimensional (2D) array of classification accuracy values representing the extent to which reactivation predicted a decision on each trial.

During planning, collapsing across both trial types, we found no evidence for reactivation of terminal states, which we would expect if reactivation supported model-based planning that depends on a consideration of nonlocal states. However, on the basis of prior work (*8*, *11*, *18*), we reasoned that the degree of reactivation might relate to the level of model-based inference needed to make successful decisions on generalization trials. Here, reactivation is likely when making plans based on outcomes that were not the object of direct experience in relation to these arms. This predicts greater evidence for terminal-state reactivation in participants who use a model-based strategy and, as detailed in Fig. 2C, participants’ behavior varied in the extent to which they deployed a model-based inference.

**Fig. 4:**
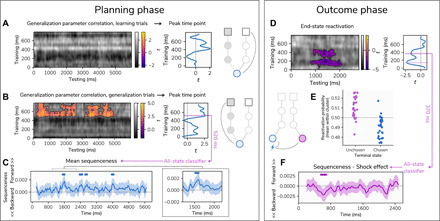
Replay and reactivation of task states. (**A**). Association between classifier prediction accuracy during learning trials and the *G* model parameter, a measure of model-based inference on generalization trials, indicating no significant associations. (**B**) At planning, the association between classifier prediction accuracy during generalization trials and the *G* parameter from our computational model. Two significant clusters are seen, one from 140 to 2860 ms and one from 3150 to 5110 ms, indicating that a late component of the subsequently chosen terminal-state stimulus representation (peaking at 520 ms) was reactivated during planning to a greater extent in participants using a model-based inference. (**C**) Replay analysis during planning, showing mean sequenceness across all paths and trials (representing evidence for sequential replay) within the trial, based on a classifier trained at 520 ms after stimulus onset. Positive values represent forward replay, while negative values represent reverse replay. The shaded region represents the 99.9% HPDI from a Bayesian regression model, and circular marks above the figure series indicate individual time points where the interval excludes zero. (**D**) Evidence for reactivation of end states during outcome phase, showing that the terminal state of the unchosen path is reactivated. Negative values represent classifier accuracy below chance, indicating that the classifier trained is predicting the opposite state to that chosen. Two components of the stimulus representation are reactivated, peaking at 150 and 370 ms. (**E**) Mean reactivation probability across the cluster shown in (D) for end states of chosen and unchosen paths during outcome phase. (**F**) Mean sequenceness using a classifier trained on the 370-ms stimulus component, where negative values indicate that shock outcomes are associated with greater reverse replay (no significant effects were seen with a classifier trained on the 520-ms component). Circular markers above the figure series indicate individual time points where the 99.95% HPDI excludes zero.

To test our hypothesis of greater reactivation in those deploying a model-based strategy, we examined the correlation between the predictive accuracy of our classifier (representing evidence for reactivation of the corresponding terminal states) and the generalization parameter derived from our computational model. The latter provides a metric of the extent to which participants used model-based inference when generalizing from learned to unlearned arms. Likewise, we reasoned that a positive association between model-based inference and reactivation should be more pronounced when a model-based inference is required. Thus, we separately conducted the analysis for learning trials (where no model-based inference is required) and generalization trials (where model-based inference is required). As expected, no significant clusters were present when we examined learning trials (Fig. 4A). Critically, for generalization trials, we found a positive association between individual generalization and choice classification accuracy during the planning period (cluster *P* = 0.007; Fig. 4B). This reflected reactivation of a component present 520 ms after stimulus presentation during the localizer task. However, an important caveat here is the fact that the interaction between learning and generalization trials was not significant.

Next, we asked whether evidence for reactivation in generalization trials embodies a sequential replay of task states, assessed using a metric termed sequenceness (*13*). To address this, we took the series of 14 classifiers, each built to distinguish an individual state from all other task states. Performance of the classifier, alongside the approximate spatial distributions of classifier weights, is shown in figs. S1 and S2. In contrast to the previous analysis, where we focused on preferential reactivation of terminal states alone, this classifier tested for reactivation of every task state, facilitating quantification of sequential replay of task state. We trained the classifiers at 520 ms after stimulus onset, based on this component representing the peak reactivation in individuals who used model-based inference to a greater extent. Applying this classifier to task data provides an index of reactivation likelihood at each time point in every trial. Using a form of temporally delayed linear modeling (*13*, *14*, *28*) of lagged cross-correlations between state reactivation vectors for different states, we then determine whether reactivations occur in a sequence consistent with task structure, where positive values indicate forward replay and negative values indicate reverse replay.

While prior studies identified evidence for sequential replay averaging across the entire trial (*15*, *18*, *28*), here, we adopted a related approach used in our prior work (*13*, *29*). This approach measures sequenceness within sliding windows that quantify fluctuations in evidence for replay within a trial enabling it to uncover time-varying patterns of replay, rather than assume a constant level of replay across the entire trial. This enables us not only to determine the presence of replay but also to characterize its temporal profile across the duration of the decision process. In essence, instead of a focus on the speed of replay (as in some prior studies), we examine the time within a trial when replay happens. This is an important characteristic to capture given that prior rodent research has shown that online replay tends to occur at a relatively consistent time before a decision point (*3*, *4*), while our prior human work also indicated that replay during decision-making tasks has a consistent temporal profile (*13*).

For this purpose, we tested for sequenceness, with state-to-state lags of up to 200 ms, within a sliding window of 400 ms across a 6-s planning period, assessing evidence for sequenceness at each time point within the trial. We used a hierarchical Bayesian latent Gaussian process regression (HLGPR) method, which accounts for a correlation between time lags (see Materials and Methods). In essence, we examined modulation of sequenceness in both learning and generalization trials by relevant task factors, assessing evidence at each time point within the trial based on the highest posterior density interval (HPDI) of the recovered Gaussian process (GP), an approach similar to that used in our prior work on time-varying replay (*13*).

On average, across trial types, all states in a path were replayed in a forward direction throughout the trial, with evidence for forward replay peaking 1600 ms into the 6-s planning phase (Fig. 4C). We found no evidence that replay intensity differed for chosen compared to unchosen paths that met our criteria for significance (see Materials and Methods). Likewise, there was no effect of trial type that might indicate differential sequential reactivation with respect to learning and generalization trials. Thus, while there was evidence of stronger reactivation of terminal states on generalization trials, there was no evidence that generalization trials were associated with greater reactivation of paths leading to, and including, this terminal state.

### Previously unchosen paths are reactivated during aversive learning

Prior work has proposed that state reactivation after feedback facilitates an outcome credit assignment that underpins value learning (*30*, *31*). Here, we examined this question in the aversive domain, using the nonsequential reactivation measure associated with generalization during planning. As at planning, this outcome phase analysis first involved training classifiers to discriminate between terminal states before using these classifiers to predict the chosen terminal state. Here, we reasoned that if representations of chosen terminal states are being reactivated following an outcome, then this should provide sufficient information for a classifier to retrospectively determine which path had been chosen on the current trial. Such a finding would indicate that the experienced state-action-outcome association was reactivated, putatively supporting credit assignment. In contrast, negative predictive accuracy would suggest that the unchosen terminal state was reactivated, supporting counterfactual value updating.

We found that the accuracy of a classifier’s predictions in a cluster starting 800 ms after outcome was significantly below chance (cluster *P* = 0.004; Fig. 4D). This is consistent with a classifier reliably indicating that an unchosen terminal state was reactivated following an outcome. The cluster included peaks evident at 370 and 150 ms in the stimulus-locked functional localizer activity, indicating that both early and late components of the stimulus perceptual representation were reactivated, a finding reminiscent of what we previously reported (*10*).

We next asked whether postoutcome reactivation embodied a sequential component, testing whether elements of the path leading up to the terminal state were replayed in sequence. We again used a sliding window sequenceness analysis to identify periods in the trial where task states were reactivated in a sequence consistent with task structure. As we observed reactivation of two stimulus components (one at 150 ms and one at 370 ms), and prior work suggests that both early and late stimulus components represent distinct features of a stimulus (*10*), we independently trained classifiers at these time points and evaluated evidence of sequential replay based on both, using a more conservative threshold to account for the two tests (i.e., controlling for multiple comparisons). There was no evidence of sequential replay, on average, across shock and safety outcomes using either the 150- or 370-ms classifier. However, average effects can conceal heterogeneity in the level of replay across trials. Decomposing this average effect on replay intensity by outcome type (shock versus safety) highlighted negative effects at several time points within the trial when using the 370-ms classifier (Fig. 4F), reflecting supportive evidence for stronger reverse replay following shock compared to safety at a cluster of time points beginning 600 ms after outcome. Notably, evidence for replay was not related to whether the path was chosen or unchosen, suggesting that shock-related replay was not specific to either arm.

### Medial temporal lobe theta power is linked to reactivation strength

Having established the presence of task state reactivation, we next sought to localize source regions supporting this reactivation. Given the importance of the hippocampus in both reactivation and replay (*15*, *17*, *18*, *32*), and the role of hippocampal theta oscillations both in memory retrieval (*33*, *34*) and avoidance (*35*), we examined whether trial-by-trial theta was associated with strength of reactivation across trials. We first performed source localization using beamforming (*36*) to provide localized estimates of whole-brain theta power over the course of each trial. For the planning phase, this was limited to generalization trials as this was where we found evidence of reactivation. After extracting mean theta power estimates from the hippocampus, we then used linear regression to predict hippocampal theta power from reactivation strength, quantified by taking probabilistic predictions from our classifiers on every trial, averaged within clusters showing evidence of reactivation (shown in Fig. 4, B and D), and performed separately for planning and outcome phases (see Materials and Methods). We performed this analysis at increasing levels of granularity, beginning by assessing predictive strength when averaging across the entire trial and then examining individual time points within the trial.

**Fig. 5:**
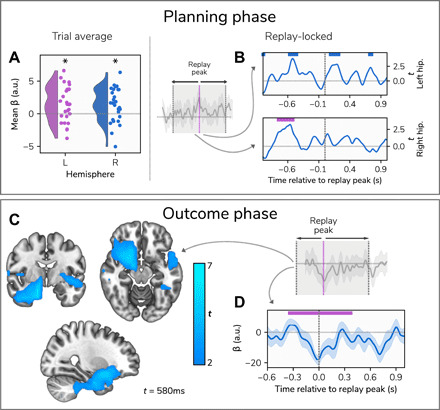
Source localization at planning and outcome phases. (**A**) Averaging across the entire trial, generalization trials showed bilateral hippocampal theta power coupled to reactivation strength during the planning phase. The figure shows all participants’ mean β weights across the trial for the effect of reactivation on theta power, which was significantly greater than zero in both hippocampi. *P = 0.01 and 0.02. a.u., arbitrary units. (**B**) The temporal profile of associations between reactivation strength and hippocampal theta power, within a 2000-ms window centered on the time point where we observed strongest evidence of forward replay (1600 ms following trial onset). The time courses represent the effect of reactivation strength on theta power across the trial, with nonsignificant clusters represented by blue markers above and significant clusters represented by purple markers. (**C**) Results of the whole-brain analysis in the outcome phase, also performed within a window centered on the time point showing strongest evidence of replay (600 ms following outcome receipt), showing a single significant cluster where theta power was negatively associated with reactivation of the chosen end state (and hence positively associated with reactivation of the unchosen end state). The image is focused on the time point 580 ms after an outcome was displayed. (**D**) Mean time course of β weights at the peak of the significant whole-brain cluster across the trial, with the replay peak indicated by a vertical dashed line and the temporal extent of the cluster shown in purple.

In the planning phase of generalization trials, we found that hippocampal theta power was predicted by reactivation strength in both the left [*t*(27) = 3.01, *P* = 0.01] and right [*t*(27) = 2.71, *P* = 0.02; Fig. 5A] hippocampi. We followed up on this by asking whether activity at specific time points during the trial was especially predictive of reactivation strength. We focused on a 2000-ms time window centered on the time point where we observed strongest evidence of forward replay (1600 ms after trial onset), with the aim of detecting points where hippocampal activity preceded or followed replay events. Here, we identified a cluster extending in time from 850 to 1090 ms after trial onset where reactivation was predictive of theta power in the right hippocampus (*P* = 0.02; Fig. 5B). When we repeated this analysis at the whole-brain level, we found no significant clusters.

Next, we examined the relationship between hippocampal theta and reactivation at the outcome phase. In the hippocampus, we found no evidence for theta power predicting reactivation strength, with nonsignificant effects across both hippocampi found when averaging across the entire trial (left *P* = 0.15, right *P* = 0.12). As with the planning phase, we next tested for significant time periods within a window centered on the time point showing strongest evidence of replay associated with shock (600 ms after outcome). There were no significant time periods within the trial where hippocampal theta power was significantly associated with reactivation strength. We also performed an exploratory analysis at the whole-brain level to identify regions outside the hippocampus where theta power was associated with reactivation strength. This identified a significant cluster encompassing the amygdala, striatum, and anterior aspects of the hippocampus and extending forward to encompass the ventromedial prefrontal cortex (vmPFC) where reactivation strength was negatively predictive of theta power in these regions (*P* = 0.01; Fig. 5C). Thus, at outcome, greater theta power in this cluster predicted stronger reactivation of an unchosen state that persisted in time from 260 to 970 ms after outcome, peaking at 580 ms, 20 ms before the time point showing strongest evidence of replay (Fig. 5D). Whole-brain *t*-statistic images are available to download at https://osf.io/rxq6z/. We also conducted additional exploratory source localization analyses within the low-gamma (30 to 60 Hz) and high-gamma (120 to 200 Hz) bands but found no significant effects.

While this demonstrated evidence of a link between theta power and state reactivation across trials, we also investigated how theta related to the temporal profile of reactivation and replay within a trial, again focusing on the a priori hypothesized hippocampal region of interest. To achieve this, we constructed separate autoregressive linear models predicting theta amplitude and phase from prior time points, in addition to reactivation and replay strength. These models revealed a significant association between theta amplitude and reactivation strength across both left and right hippocampi and showed a similar relationship with replay intensity in the right hippocampus alone. No significant effects were seen with respect to theta phase. Additional analyses revealed a limited effect in the low-gamma band (30 to 60 Hz), where higher-gamma amplitude was associated with greater reactivation. Full results are reported in table S2 and fig. S5.

Last, we examined relationships between the *G* parameter from our behavioral model and both hippocampal theta power and the coupling between hippocampal theta power and reactivation and found no significant associations at either planning or outcome phases. Full results are shown in the Supplementary Materials.

## DISCUSSION

We show that task states are replayed during aversive learning in a manner that supports model-based planning and value updating, consistent with a simulation of trajectories where direct experience of the consequence of choosing a trajectory is unavailable. These findings, consistent with rodent work, highlight a role for reactivation and replay in learning and planning during avoidance in the absence of direct experience.

During planning, a preferential reactivation of task goal states was seen when model-based inference was required. This, in turn, depended on the extent to which individual participants actually relied on a model-based strategy. While a difference between learning and generalization trial types was not significant, the significant correlation of reactivation during generalization with individual differences in model-based inference provides tentative evidence that use of this strategy may rely on prospective reactivation. Our finding here is reminiscent of previous work in the reward domain showing that reactivation supports decisions based on model-based inference of state-value associations in participants who demonstrate behavior consistent with this type of strategy (*8*, *11*).

Reactivation strength during planning, in trials requiring model-based inference, was positively associated with hippocampal theta power, with the strongest effect occurring within 1 s of trial onset, suggesting that reactivation may be hippocampal dependent, as has been reported in rodents (*37*, *38*). Such reactivation occurred as part of a pattern of sequential reactivation of task states in a forward direction, as also found in rodents (*39*). This implicates reactivation during avoidance planning when direct experience of outcome receipt following a specific state transition is unavailable, consistent with a simulation of outcomes and paths for actions that are yet to be taken.

After outcome receipt, reactivation of the unchosen outcome state was complemented by replay analyses where, following shock outcome, path sequences were replayed in a reverse order. Notably, replay was not specific to either the chosen or unchosen path, indicating that while unchosen terminal states were preferentially reactivated, reactivation of paths leading to unchosen terminal states was not necessarily more sequential in nature. Postoutcome reactivation has been proposed as a mechanism through which credit is assigned to states preceding an outcome (*31*) and through which cognitive maps are updated (*16*, *40*). We found that the reactivation of unchosen states was associated with theta power in a cluster encompassing medial temporal lobe (MTL) and in an exploratory analysis with regions that encompassed the amygdala, vmPFC, among others. While we cannot pinpoint the precise source of this reactivation using MEG alone, the regions within this cluster include those proposed to represent cognitive maps across multiple task domains (*41*, *42*). These regions are implicated also in counterfactual thinking (*43*–*45*) and avoidance (*35*, *46*, *47*). Our results extend upon this prior work and would be consistent with a proposal that these regions support a form of counterfactual updating based on a learned model of the environment. This updating occurred rapidly after trial onset, at approximately the same time as the emergence of evidence for reverse replay, implying that these regions might initiate reactivation of relevant task states after outcome receipt, although our analyses cannot confirm this.

Our results have parallels to findings in rodents showing preferential awake replay of paths leading to conditioned shock zones before avoidance (*4*). We find that terminal states for chosen, rather than avoided, paths are reactivated. Aside from species differences, in our task, participants made deliberative avoidance plans that relied on model-based knowledge. In contrast, the prior study focused on reactive movements away from threat during free navigation (*4*). Nevertheless, our results hint that similar mechanisms may support human model-based avoidance, implicating reactivation in retrieving and maintaining representations of paths toward safety and facilitating avoidance of locations associated with threat. Future work will be required to understand factors that bias reactivation toward chosen or unchosen paths in humans and other animals. In addition, while we found evidence of sequential replay during planning, its strength was only weakly related to decisions or task factors, implying that sequential replay may provide a readout of the task structure regardless of behavior or current task phase, as suggested by prior work in humans (*14*).

Our work provides evidence of prospective and retrospective reactivation and replay during planning and learning in aversive environments in humans. Growing evidence implicates reactivation (*8*, *10*, *11*, *48*) and replay, measured by both functional magnetic resonance imaging (*17*) and MEG (*14*–*16*, *18*), during learning and decision-making in the reward domain. However, despite being critical for understanding common symptoms of psychopathology, the aversive domain has received little attention. One study has reported alternating reactivation of potential action outcomes during avoidance decisions (*19*), suggesting that the brain simulates outcomes of its actions during deliberation, though this latter work did not address state reactivation nor model-based computation, which is the prime focus of the current work. This is a subtle, but important, distinction as reactivation of environmental states during model-based control requires an explicit internal model of these states and the transitions between them. Our results point to similar mechanisms that are at play in both aversive and rewarding contexts, and it is also notable that replay in our task occurs online (i.e., during a task, as opposed to at rest) within very brief time windows, as shown in recent work using nonaversive tasks (*16*, *18*).

The approach that we used allowed us to identify specific time windows wherein task states are replayed in sequence. Echoing previous results (*13*, *29*), we found bursts of sequential reactivation at consistent time points within the trial. During planning, these peaked at 1600 ms into the decision-making phase and at 600 ms following shock outcome receipt, suggesting that sequential replay is not necessarily an ongoing process but one that occurs at specific times. This may reflect the fact that the task used here is relatively simple in terms of planning requirements, involving relatively small state and action spaces. In more complex tasks, state reactivation might persist across time to facilitate ongoing planning (*14*). Another notable feature of our results is the reactivation of a relatively late component of the perceptual stimulus response (peaking at 520 ms after onset in the planning phase and 370 ms in the outcome phase), which is reminiscent of prior work involving sensory preconditioning (*10*), and where this later reactivated component was linked to representations of stimulus category (e.g., faces or scenes) rather than immediate perceptual characteristics (*10*). Although our task was not designed to dissociate different levels of stimulus representation, this timing suggests reactivation of a similar category-level representation in the present study. In addition, we note that classifier weights were strongest in occipital and temporal sensors, indicating that we are likely detecting reactivated stimulus representations in visual cortex or downstream temporal regions. We speculate that this does not reflect the source of reactivation [which is presumed to be hippocampal in origin, as suggested by our source localization results in addition to prior work in both rodents and humans (*3*, *15*, *18*)], but instead regions wherein perceptual qualities of a reactivated state are represented and reinstated.

A limitation of our task is its inability to disentangle choice and threat perception, as participants’ decisions are guided by a desire to avoid threat, and this entails that unchosen sequences are avoided precisely because they are perceived as potentially leading to negative outcomes. This means that we cannot determine whether reactivation is prioritized by value or by the decision-making process itself. In addition, our task to some extent confounds value and choice with experience. Aversive, unchosen, paths are inevitably experienced less than safe paths, and it is possible that they are preferentially reactivated as a way to equalize experience. However, our task was designed to minimize this possibility as each path is preferable on the same number of trials, meaning that participants choosing optimally should experience both paths approximately equally. The task used in this study also involved an explicit requirement for participants to consider the sequential nature of the states. It is possible that the sequential replay that we observe arises as a direct by-product of this instruction, rather than reflecting a naturalistic phenomenon. However, we believe that this is unlikely given the wealth of research in animals showing that replay events occur spontaneously in less constrained environments, without the possibility of formal instructions (*3*). Furthermore, the speed of sequential neural replay (<200 ms between states) appears much faster than an explicit planning process. Last, our measure of replay relies on the subtraction of forward from reverse replay to account for the autocorrelation in state reactivation patterns (*14*, *15*), such that our replay measure represents a dominance of either forward or reverse replay rather than either independently.

Reactivation may be recruited to simulate state transitions and outcomes that have not, or cannot, be physically experienced. This is the case, for example, when making decisions based on transitions that cannot be directly experienced or when updating value estimates of states that were avoided. This process may be relevant to understanding psychiatric symptoms that have been linked to reactivation of negative memories, most prominently worry and rumination seen in anxiety and depression (*7*). Notably, the content of worry and rumination is often counterfactual in nature, focusing on actions not taken (*49*, *50*), providing at least a conceptual link to the postoutcome results reported here. More speculatively, our exploratory analysis implicated a wide set of regions that included MTL, vmPFC, and related areas in an initiation of state reactivation, and these include regions implicated in depression (*51*–*54*). By establishing a role for neural replay in aversive learning, our results provide a foundation for future work that enables an examination of how aversive neural reactivation and replay may relate to specific symptom characteristics seen in states of anxiety and depression.

## MATERIALS AND METHODS

### Participants

We recruited 28 participants from University College London (UCL) participant databases who provided informed consent before beginning the study. The subjects were paid at a rate of £10 per hour, plus up to an extra £10 bonus for performance during the functional localizer task. The study was approved by the UCL Research Ethics Committee (reference 12707/002).

### Functional localizer

The first task performed by the participants in the scanner was a functional localizer task designed to elicit neural responses specific to each stimulus used in the main task. Each stimulus consisted of an image showing an everyday object, such as a key or a house (images are shown in fig. S3). The same set of images was used for all participants, but the pairing between images and task states was randomized. The purpose of this was to provide data to train classifiers to decode reactivation of each image during the task itself. Each of the 14 images used in the task was shown for 0.25 s, followed by two words shown on either side of the screen. Images were randomly presented for each participant over the 900 trials, ensuring that each participant saw each image approximately 64 times, although the precise number of presentations varied slightly. One of these words described the previously shown image, and the participants had to select the correct one by pressing one of two buttons on a keypad. Following this decision, there followed a 0.25 (± 0.15)–s intertrial interval before the next trial beginning. To boost motivation and encourage attention to the task, the participants were given a bonus payment of up to £10 that was dependent on their accuracy. The subjects completed 900 trials over six blocks.

### Task

The subjects completed an aversive reversal learning task in which they were instructed to avoid mild electric shocks. The task involved navigating through a map comprising 14 unique states, each state represented by a visual image (Fig. 1A). The map consisted of four arms, each comprising a path of four unique states: a start state, two intermediate states, and a terminal state. Although this can be conceptualized as a spatial environment, the participants were not explicitly told of any spatial relationship between states. Each of the two end states was endowed with a probability of shock that varied according to a random walk, with the same pattern of probabilities used for every participant. Overall, each end state was safest on approximately 50% of trials, ensuring no overall bias toward one or the other being safest. The subjects were informed that these shock probabilities would change across trials and that the two end states had independent shock probabilities.

Outside the scanner, the participants first underwent a training procedure where they learned how to navigate successfully through states. They were first allowed to freely navigate through the two arms of the maze, and “valid” transitions from the current state were indicated visually. This enabled the participants to learn which sequences of images were valid and which were not. After 10 trials of this free navigation, we assessed the participants’ knowledge of the task structure. In this phase, the participants were presented with the first image (shared between both sequences) and one of the two final images. They were then able to sequentially select the intermediate images that would take them from the first to the last state. The participants progressed to the next stage if they chose correct states on 8 of 10 trials. If this was not achieved, then they returned again to the free navigation phase. This procedure repeated until the success criterion was met, ensuring that all participants had adequate knowledge of the task structure. Crucially, this pretraining used a different set of images to the real task enabling the participants to learn the task structure without contaminating the true images with structural information.

In the main task, two arms of the map were available to select on each trial (starting with states A and D or B and C; Fig. 1A). To select the arm that they wished to take, the participants were asked to select a single state in the arm (which could be any state from that arm) from an array of four potential states, prompted by the position of that state in the sequence. Thus, although the participants were required to know the entire sequence of states for each arm, on any given trial, they selected only one to indicate their choice. This served to shorten the selection time while ensuring that the participants maintained an ordered representation of the state sequences, rather than representing the arms through their start states alone. For example, given a choice between arm A > E > I > M and arm D > H > L > N (see Fig. 1A), and being shown the number 2 (indicating the second state in the sequence of states for each arm), the participants would choose either E or H (depending on their preference for either arm) from an array of four images including E and H.

After the functional localizer task, we calibrated a shock level for each participant. This was done before retraining on the task to minimize distraction. We followed a procedure used previously (*25*), whereby the participants are given increasingly stronger shocks to their nondominant hand and asked to rate their intensity on a scale ranging from 1 to 10, where 10 was the most that they would be willing to tolerate. Intensity was increased until a level of 10 was reached, and this was repeated three times. The average of these three shock intensities was recorded, and we used 80% of this level as the shock intensity during the task. Following shock calibration, the participants repeated the training procedure. This was identical to the out-of-scanner pretask training but included the real images used in the functional localizer and task and served to teach the true transitions used for the task. We again ensured that participants met the preestablished criterion for structure knowledge, as our investigation focused on the use of a prelearned map, not the learning of the map itself.

The main task is illustrated in Fig. 1. Following a 6-s planning phase, the participants selected the path that they wished to take using the same method as outlined for the training. Once the participant had selected a full sequence of states, the sequence was played back at 1.6-s intervals (1.2 s for the final state). After 1.2 s where the final state was displayed, the outcome was shown on an otherwise blank screen for 2.8 s. The type of outcome was signaled by the presence of a shock symbol (for a shock outcome) or a crossed-through shock symbol (for a no-shock outcome).

The critical additional manipulation was a distinction between “learning” and “generalization” trials. In learning trials, the participants chose between the learning arms of each of the two mazes. Having made a decision, they were shown the full sequence of states leading to the end state, before being shown the outcome associated with that end state. Thus, they were able to learn a shock probability associated with each of the two learning paths and behave accordingly using model-free learning. On generalization trials, they made a similar choice from two generalization arms and once their decision was made, the next trial began. On these generalization trials, the participants were deprived of a playthrough of the selected arm states and the ensuing outcome, obviating model-free learning based on direct experience. For learning trials, outcomes were accumulated within each of the five blocks, and three of these outcomes (either shock or safety) were chosen at random at the end of the block and administered, with a 5-s gap between outcomes involving shocks. Thus, at the end of each block, each participant received three outcomes, which were biased in favor of shock or safety depending on their choices during the task. For generalization trials, the participants were told that the outcomes were instead accumulated throughout the task, and 10 of these would be administered at the end of the session. However, this was not actually implemented, as there was no experimental need to administer the shocks after the task. Shocks were only administered during the task itself, as the training phase only involved structure learning rather than value learning. The task had 120 trials and took approximately 35 min.

### MEG acquisition and processing

Scans were acquired on a CTF 275-channel axial gradiometer system (CTF Omega, VSM MedTech), sampling at 1200 Hz. All preprocessing was conducted in MNE-Python (http://mne-tools.github.io/) (*55*). Eye tracking data were recorded simultaneously using an EyeLink 1000 system. Data from 272 usable channels were first Maxwell-filtered to remove noise, before being band-passed between 0.5 and 45 Hz using a finite impulse response filter. After filtering, independent component analysis was performed on the raw data to isolate noise-related components. Components related to eye blinks were detected through both correlation with eye tracking measures and visual inspection and subsequently removed from the data, while cardiac components were detected and removed using automatic tools provided in MNE. Last, data were downsampled to 100 Hz to reduce computational burden for temporal generalization and sequenceness analyses, and to 400 Hz for source localization analyses.

### Computational modeling of behavior

To investigate how replay relates to learning processes, we fit computational learning models to participants’ behavioral responses. These models were Rescorla-Wagner (*23*) variants, where value is updated on each trial (*t*) according to a prediction error (δ) weighted by a learning rate (α)

$$\delta_{t}={\text{outcome}}_{t}-V_{t}$$ (1)

$$V_{t+1}=\mathrm{Vt}+\alpha \cdot \delta_{t}$$ (2)

As the two end states had independent shock probabilities, the model separately learned the value of each, resulting in estimates of $V_{t}^{M}$ and $V_{t}^{N}$ representing the two end states. Second, as the two end states had independently fluctuating shock probabilities, we adapted the update step to allow asymmetric learning that included separate learning rates for better- and worse-than-expected outcomes (α^+^ and α^−^, respectively)

$$V_{t+1}^{M}=V_{t}^{M}+\begin{array}{c} \alpha^{+}\cdot \delta \;\text{if}\;\delta_{t}>0\\ \alpha^{-}\cdot \delta \;\text{if}\;\delta_{t}<0 \end{array}$$ (3)

We also modified the model to capture choice on generalization trials. Here, the choice was assumed to depend on the learned value of each arm’s end state (based on a generalization of experience from learning trials). However, we allowed the degree of this influence to vary between participants by modulating it with an additional parameter, *G*

$$V_{t}=0.5+(V_{t}-0.5)\cdot G$$ (4)

Here, *G* governs how much influence the learned end state value exerts on choice in generalization trials. At a value of 1, these choices are fully determined by the value of the end state and so are fully consistent (subject to decision noise) with choice probabilities on learning trials (i.e., if the participant chooses A > E > I > M on a learning trial with probability 90%, they are 90% likely to choose B > F > J > M on a subsequent generalization trial because of the shared end state). At a *G* value of 0, choices are not influenced by learned value at all, and value is unbiased (i.e., a value of 0.5 on a scale from 0 to 1). On generalization trials, values in the model were not updated as no outcome was presented on these trials. This model assumes that value is learned only for the terminal state in the sequence, and on learning trials, only the chosen stimulus is updated. Value is then converted to a binary choice probability using a softmax function with an estimated temperature parameter (β)

$$P_{t}=\frac{e^{{(\beta \cdot V}_{t})}}{e^{{(\beta \cdot V}_{t})}+e^{{(\beta \cdot (1-V}_{t}))}}$$ (5)

As choice probabilities did not sum to zero, they were normalized following this calculation.

All behavioral models were fit using hierarchical Bayesian methods using variational inference implemented in PyMC3 (*56*). For further analyses involving model-derived measures, data were simulated from the model using means from each participant’s posterior distribution over each parameter value. Prediction errors were calculated by taking the difference between the expected value from the model and value of the presented outcome (1 for shock and 0 for safety).

### Temporal generalization analyses

To perform temporal generalization analyses, we trained binary classifiers on data from a functional localizer to enable us to differentiate between neural representations associated with each of two given image stimuli. We built a classification pipeline using tools from MNE (*55*) and Scikit-Learn (*56*) that consisted of first scaling the data to have zero mean and unit variance, before performing dimensionality reduction using principal components analysis (PCA) with 50 components and then classification using L1 regularized logistic regression. The L1 regularization hyperparameter of the logistic regression classifier was optimized using random search to produce the highest possible classification accuracy, drawing 100 potential values from a half-Cauchy distribution with γ = 5. We trained classifiers to distinguish between start states and end states for both learning paths and generalization paths separately, resulting in three classifiers (one for learning paths, one for generalization paths, and one for the shared end states).

We applied these classifiers to the task data to determine whether they successfully predicted the path that the participants chose on each trial. If the representations that classifiers were trained on were reactivated during the task in a way that relates to choice (for example, reactivating state A when choosing the path that state A is a component of), then the classifiers can capitalize on this information to predict the participant’s choice. Therefore, accurate choice prediction provides evidence of reactivation of perceptual representations of task stimuli. It is important to note that the classifiers being used to predict task choices were trained on data collected before the actual task, and therefore, they are completely dissociated from any kind of value or structural associations that the stimuli acquired during the actual learning task.

### State decoding for replay analyses

Data were extracted from poststimulus onset time points based on those showing evidence of reactivation in the temporal generalization analyses. We chose this approach as a principled way of targeting specific stimulus components that we expected might be reactivated on the basis of prior analyses, enabling us to avoid multiplicity issues that arise when using a more exploratory approach. We acknowledge that it is possible that focusing on alternative time points might also reveal evidence of replay, and we cannot rule out this possibility. These analyses were conducted using tools from Scikit-Learn (*56*). Additional data were included from 50 ms either side of this time point, such that data provided to the classifier included the period between 100 and 200 ms after stimulus onset as a form of temporal embedding. Data were subsequently subject to a dimensionality reduction procedure using temporal PCA before being transposed to an 11 (the number of time points)–by–*n* 2D matrix, where *n* represents the number of principal components. These data were then scaled to zero mean and unit variance, before being submitted to a regularized logistic regression with L1 penalty. Classification was performed using a one-versus-the-rest strategy, where multiple classifiers are trained to discriminate between the stimulus of interest and all other stimuli. Sample weights were balanced to ensure that classification probability was not biased by frequency of training examples. Optimal values for the number of principal components and the L1 regularization hyperparameter of the classifier (α) were jointly optimized through 100 iterations of randomized search (*57*) to identify the settings producing the highest classification accuracy for each participant, with each iteration of the search evaluated using threefold cross-validation. The number of components was sampled from a uniform distribution unif (30, 60), while α was sampled from a half-Cauchy distribution with γ = 5.

### Replay analysis

To index reactivation of task states, during planning and task rest periods, the classification pipeline trained on each participant’s functional localizer data was applied to the data to produce a probabilistic prediction of the state being activated at each time point. This involved extracting data for 50 ms before and following the time point of interest, applying the PCA decomposition and scaling determined using the localizer data, and multiplying the data by the trained feature weights to produce a probabilistic prediction of which state was activated at the specified time point. This resulted in a matrix representing the probability of each state being reactivated at each of 590 time points (the 600 available time points minus the first and last 5 time points, which are lost because of the temporal embedding procedure).

To detect sequential reactivation of state representations in these data, we used a cross correlation method (*13*, *14*, *29*). While this method does not allow us to investigate forward and reverse replay independently because of the high autocorrelation present in the signal (instead, we use the difference between these metrics to obviate this autocorrelation), the cross-correlation method is more amenable to windowed analyses than general linear modeling approaches (*28*). Here, we took the product of the state × time reactivation matrix and the task transition matrix, where the time course of each state’s reactivation probabilities was correlated with the time courses for all other states, lagged by up to 20 time points (representing 200 ms) in steps of one. This produced a matrix of correlation coefficients representing the effect of past reactivations (across 20 lags) on current state reactivation and, hence, the correlation between past activation and present activation, within the transition matrix of the task. This allowed us to isolate replay of transitions that were consistent with the transitions in the task (representing forward replay) or reverse transitions (representing backward replay). These were then subtracted to produce a difference measure representing the balance of forward versus reverse replay. We assessed the transition matrices representing each arm separately, resulting in vectors of evidence for replay (termed sequenceness) across each time lag (up to 200 ms) for each arm. To assess how sequenceness fluctuates within trial, we performed this procedure within 400-ms sliding windows, as in prior works (*13*, *29*). This provided an index of sequenceness at each time point within the trial.

### Statistical analysis of sequenceness data

Our sequenceness detection procedure provided a measure of sequential replay at each time point, within each trial, for each participant. Our aim was to identify periods during the trial where we observe consistent evidence for sequential replay across participants and examine whether replay is modulated by task factors (such as path type and choice). To achieve this, we used HLGPR. This builds upon prior work using hierarchical GPs to directly model observed time series, both in MEG work (*13*) and other fields (*58*, *59*), and provides an intuitive approach to modeling time series data while accounting for its covariance structure. This is particularly appealing for the analysis performed here, as directly accounting for covariance allows us to circumvent multiple comparison corrections across time points, which presents a problem when identifying significant effects if we expect these effects to be brief in their duration.

Here, we extend on this prior implementation to allow more flexible analysis of time-varying modulation of the observed time course. Rather than directly representing the observed time series as a GP, the HLGPR approach instead represents the observed time series as a linear combination of input features and time-varying regression weights, which are modeled as latent GPs. As these latent GPs represent a distribution over functions, we can use Bayesian modeling to approximate the posterior distribution over potential functions linking task features to replay over time.

We define this as a Bayesian linear model, where the observed data at each time point *t* are modeled as a normal distribution with mean defined as the combination of regressors and regression weights at each time point and a single estimated variance across all time points σ

$$y_{t}\sim N(\beta_{t}x,\sigma )$$ (6)

The mean of this distribution is calculated using a standard linear regression model for regressors 1, …, *n*

$$y_{t}=\beta 0_{t}+\beta 1_{t}\cdot x1+\ldots +{\beta n}_{t}\cdot \mathrm{xn}$$ (7)

Here, each β*_t_* is one point on a function described by a latent GP, resulting in regression weights being represented as GPs of the same length as *y*, one per regressor plus an intercept. The regressors themselves are assumed to be constant for the duration of the trial (representing a variable such as trial number, or choice on the trial). We then extend this model to allow both random slopes and random intercepts, whereby observed data for each time point *t* within each participant *i* = 1, …, *n* participants is represented by the following regression model

$$y_{t,i}\sim N(\beta_{t,i}x_{i},\sigma )$$ (8)

$$y_{t,i}=\beta 0_{t,i}+\beta 1_{t,i}\cdot x1_{i}+\beta 2_{t,i}\cdot x2_{i}+\ldots +{\beta n}_{t,i}\cdot xn_{i}$$ (9)

This results in a separate function per each participant for each regressor, allowing the time course of each regressor’s effect to vary across participants. However, rather than treating each participant independently, we represent the data using a hierarchical model where we account for covariance between participants. This approach allows us to take advantage of the benefits of hierarchical models in terms of aiding parameter estimation when using data that are limited in quantity and quality, as is typical of neuroimaging data. To achieve this, we turn to prior work on hierarchical GPs (*58*, *59*) and use a hierarchical mean and covariance structure to define the GPs used to represent regression coefficients. For each regressor, we first define a constant mean group-level GP representing the group mean time-varying regression weights. For consistency with prior work, we use *x* to represent each time point

$$g(x)\sim GP(\mu ,k_{g}(x,x_{⊺}))$$ (10)

The covariance function *k_g_*(*x*, *x*_⊺_) is flexible, depending on the expected covariance structure of the data. Here, we used the squared exponential kernel, which is parameterised by a length-scale parameter 𝓁_group_, for which we use a gamma prior (γ(3,1)). We also estimate the variance of the kernel using an additional free parameter σ_group_, which is given a gamma prior (γ(3,5))

$$k(x,x_{⊺})=\sigma^{2}\;\text{exp}[-\frac{{\left(x-x_{⊺}\right)}^{2}}{2L^{2}}]$$ (11)

We place a normal prior 𝒩(0,5) on the mean at the group level (μ), allowing the regressor to have a constant effect across all time points within the trial. We assume that each participant’s function for each time-varying regression weight is offset from this by a degree determined by a second, participant-level offset function, modeled as another GP. Thus, each participant’s regression weights are represented as the summation of the group-level function and their own participant-level function. This participant-level function represents the degree to which their own function is offset from the group function, rather than representing the participant’s own function itself. This is defined as a GP with mean *g*(*x*), representing the group-level effect, and a participant-level covariance function, for which we again use a squared exponential kernel with an estimated participant-level variance parameter σ_participant_ and length scale parameter 𝓁_participant_

$$f_{i}(x)\sim GP(g(x),k_{f}(x,x_{⊺}))\;i=1,\ldots ,\text{participants}$$ (12)

As GPs are nonparametric, the only parameters that we estimate are the length-scale parameters of the covariance functions. For conceptual simplicity and computational ease, we assume the same length-scale parameter across participant- and group-level GPs but allow this to vary across regressors.

This approach provides us with group-level GPs that represent the effect of a given regressor on sequenceness at each time point in the trial. This approach allows a far greater amount of input data to be considered than modeling time series as observed GPs. For example, here, we are able to use sequenceness time series for each trial, rather than averaging across trials as in prior work (*13*). This both provides a far richer source of input data and allows us to explore the effects of continuous regressors. Using a hierarchical formulation also allows us to take advantage of the properties of hierarchical models that permit more accurate parameter estimation, while taking a Bayesian approach allows us to approximate the full posterior over parameters and, hence, the posterior over functions. As a result, we can extract both the estimated mean effect of a given regressor and the 99.9% HPDIs at each time point as a conservative range of likely values for the true function, allowing us to judge whether we can be confident that it has a nonzero effect at any given time point without the need for multiple comparison correction. For analyses in the outcome phase, we used the 99.95% HPDI to account for the two time points used to train classifiers. Validation analyses are presented in the Supplementary Materials, demonstrating that this method successfully controls the false-positive rate. However, to provide additional protection against false positives, we only interpret time points that were part of a cluster of three or more temporally contiguous time points with HPDIs that did not include zero.

For the planning phase, we included regressors representing trial type (learning or generalization), path type (learning or generalization); whether the path was chosen; the interaction between trial type and path type; and trial number. For the outcome phase, we included regressors representing whether the path was chosen, the path type, the outcome (whether or not a shock was received), the absolute prediction error, the interaction between outcome and absolute prediction error, and the trial number.

These models were constructed and fit in Python using PyMC3 (*60*), and we approximated posterior distributions of model parameters using variational inference. To ease the computation burden of this analysis we decimated the data by a factor of 4, reducing our effective sample rate from 100 to 25 Hz.

### Source localization analysis

Source localization analyses were performed using the MNE-Python implementation of the unit noise gain linear constrained minimum variance beamformer method. Data from task periods of interest (planning and outcome phases) were first filtered between 4 and 8 Hz to retain only activity in the theta band using a finite impulse response filter. The head model used to generate the source space was the standard FreeSurfer subject (“fsaverage”) with a 5-mm grid, and the forward solution for each participant was calculated using this head model scaled according to participant-specific transform parameters, determined manually by aligning participants’ head position coils with those of the standard subject. The covariance of the data was estimated on the basis of the full duration of the trial, while noise covariance was estimated on the basis of sensor data from 500 ms before outcome to outcome onset (this was used for both planning and outcome phases). Covariance matrices were regularized by a factor of 0.05. This procedure provided source activity estimates for each epoch. As we were interested in theta power, we finally applied a Hilbert transform to the source estimates (already restricted to the theta band) to provide an index of instantaneous power in the theta band. In additional exploratory analyses, we repeated the analyses focusing on low gamma (30 to 60 Hz) and high gamma (120 to 200 Hz). We chose to focus on the bilateral hippocampus and, therefore, extracted mean power time series from these regions (defined according to the FreeSurfer atlas) for our primary analyses. However, we also performed exploratory whole-brain voxel-level analysis to identify effects outside our regions of interest.

### Source-level prediction of reactivation strength

To identify associations between theta power at the source level and reactivation strength, we used a general linear model predicting theta power from reactivation strength across trials, in addition to an intercept term, at each time point within the trial. Reactivation strength was calculated using probabilistic predictions from our classifiers trained to distinguish between states of interest in our temporal generalization analyses. These probabilistic predictions can be interpreted as providing an index of the strength of reactivation on a given trial (for example, if the classifier predicts that state A is reactivated with 90% probability, then we take this as strong evidence for reactivation of state A). As we train classifiers at multiple time points in the functional localizer and apply them across multiple time points in the task, we generate a matrix of predictions for each trial. To obtain a single value per trial indicating strength of reactivation, we extract the mean predicted reactivation probability within a region of interest based on clusters showing significant effects in the temporal generalization analysis. The linear model was then constructed as follows

$$\begin{array}{cc} {\text{theta power}}_{t,i}= & \beta 0_{t,i}+\beta 1_{t,i}\cdot x1_{i}+\beta 2_{t,i}\cdot x2_{i}+\ldots +\\ & {\beta n}_{t,i}\cdot xn_{i} \end{array}$$ (13)

where *t* represents the time point in the trial, *i* represents the trial number, and *x* represents a regressor. For the outcome phase, we additionally included regressors representing the outcome of the trial (shock or safety), the absolute prediction error (derived from the computational model), and the interaction between outcome and absolute prediction error (representing a signed prediction error). All regressors were mean-centered before being entered into the model. This resulted in a time series of β coefficients for each regressor at each time point in the trial. This procedure was performed for each participant using mean extracted theta power from both hippocampi and at the voxel level in whole-brain analyses. Before aggregating results across participants, we first standardized the β coefficients by dividing them by their variance to ensure that they remained on a consistent scale.

We assessed significance in three steps. First, we examined the average association between theta power and reactivation across the trial by taking the mean of the β time series for each participant. These mean β values were then compared against zero using a one-sample *t* test, with *P* values subject to Bonferroni correction for two comparisons (left and right hippocampi). Second, we sought to identify periods in the trial where theta power was most strongly associated with reactivation. Here, we used one-sample permutation tests on β time series against zero with sign flipping to identify significant clusters in time (based on cluster mass). Third, we examined whole-brain results using 4D cluster–based permutation tests to identify significant clusters across space and time. Beta maps were first thresholded at *P* < 0.01 voxelwise to identify clusters, which were then tested for significance using permutation methods based on their mass across space and time.

In addition to these analyses using averaged reactivation, we also examined relationships between hippocampal source signals and reactivation, in addition to sequential replay, within trial. We performed these analyses using amplitude and phase as dependent variables in separate models. This involved first extracting the mean signal from the hippocampus in the three frequency bands of interest (theta, low gamma, and high gamma). Next, autoregressive linear models were constructed, predicting amplitude or phase on a given time point from the five preceding time points in addition to the corresponding state reactivation or sequenceness time course at the same time point. This enabled us to investigate the extent to which reactivation and replay were coupled to hippocampal power or phase while controlling for a temporal autocorrelation of these signals. These models were estimated at the participant level, and β coefficients were then tested for significance at the group level using one-sample *t* tests, with *P* values corrected for multiple comparisons using false discovery rate correction.

## SUPPLEMENTARY MATERIALS

Supplementary material for this article is available at http://advances.sciencemag.org/cgi/content/full/7/31/eabf9616/DC1

## Appendix

View/request a protocol for this paper from *Bio-protocol*.
